# Thyroid dysfunction in elderly first-episode drug-naïve major depressive disorder patients with comorbid dyslipidemia: prevalence, clinical profile and associated factors

**DOI:** 10.3389/fpsyt.2025.1742901

**Published:** 2026-01-12

**Authors:** Xiuhua Song, Lei Yi, Shenghai Wang, Xiangyang Zhang

**Affiliations:** 1 Department of Psychiatry, Mental Health Center of Qingdao City, Qingdao, Shandong, China; 2 Hefei Fourth People’s Hospital, Anhui Mental Health Center, Affiliated Psychological Hospital of Anhui Medical University, Hefei, China

**Keywords:** major depressive disorder, lipid metabolism, thyroid hormones, first-episode, HAMD score

## Abstract

**Background:**

Elderly major depressive disorder (MDD) patients often exhibit complex symptomatology and high rates of abnormal lipid metabolism (ALM), yet predictors of abnormal thyroid function in those with co-occurring lipid metabolism are not fully elucidated. This study aimed to investigate the relationship between thyroid function and ALM in first-episode, drug-naïve (FEDN) elderly MDD patients.

**Methods:**

1,029 elderly FEDN MDD patients were enrolled. Demographics, lipid profile (TC, TG, LDL-C, HDL-C), thyroid parameters (TSH, FT3, FT4, TG-Ab, TPO-Ab), and symptom ratings (HAMD, HAMA, PANSS-positive) were assessed.

**Results:**

Prevalence of abnormal thyroid function was 69.0%. Compared with ALM patients without thyroid dysfunction, those affected had higher HAMD, HAMA, and PANSS-positive scores, more suicide attempts, severe anxiety, and psychotic symptoms (all P < 0.05). Logistic regression identified BMI (OR = 1.186), HAMD score (OR = 1.201), blood glucose (OR = 4.03), TC (OR = 1.683), and LDL-C (OR = 1.299) were independent associated factors for abnormal thyroid function in MDD patients with comorbid ALM(all P < 0.001).

**Conclusion:**

Abnormal thyroid function is highly prevalent in elderly FEDN MDD patients with ALM; clinical severity, glucose, and lipid levels contribute to this comorbidity. These findings suggest that elderly, first-episode MDD patients with dyslipidemia, particularly those with elevated BMI, high blood glucose, and severe depressive symptoms, should undergo routine screening for thyroid dysfunction to facilitate early intervention.

## 1 Introduction

As a highly prevalent mental disorder (1, 2), major depressive disorder shows rising global incidence in elderly populations, with prevalence estimates ranging from 10% to 15%(95% CI: 10.5–16.1%)in this demographic (3). Chinese longitudinal research confirms that comorbid chronic conditions significantly increase depression risk in adults aged ≥45 years (4). Major depressive disorder (MDD) is associated with an elevated incidence of metabolic syndrome, with dyslipidemia being a frequently observed clinical manifestation that becomes more pronounced with advancing age. A growing body of evidence indicates a correlation between depression and abnormal lipid metabolism (ALM) (5). For instance, Enko et al. reported that depressed patients had elevated triglyceride (TG) and reduced high-density lipoprotein (HDL) cholesterol levels compared to healthy controls (6). The field of lipidomics has shown promise as a tool for detecting psychiatric conditions such as depression (7). Although lipid abnormalities are common in MDD patients, study findings have been inconsistent. For example, while Wei et al. documented higher TG and lower HDL-C levels in Chinese first-episode MDD patients—with no significant differences in LDL-C and TC levels (8)—Bharti et al. found increased TG but decreased TC in MDD patients, and lower HDL-C specifically in those over 40 (9). In contrast, Bot et al. observed higher TG and lower HDL-C consistently across age groups (10). These divergent results may stem from variables such as ethnicity, age, and medication use. Furthermore, prior studies indicate that antidepressant treatment can ameliorate lipid profiles, thereby reducing hyperlipidemia risk in MDD patients (11). Consequently, further investigation is warranted to clarify the status and determinants of ALM in MDD.

Thyroid dysfunction is a common co-morbidity in MDD, with both hypo- and hyperthyroidism increasing depression risk (12, 13). In MDD patients, subclinical hypothyroidism correlates with suicide risk and psychotic symptoms (14), and thyroid parameters are linked to suicidal ideation (15).

A bidirectional interaction exists between thyroid and lipid metabolism: thyroid dysfunction (especially hypothyroidism) can cause hypercholesterolemia via decreased LDL receptor activity and impaired T3-mediated control of SREBP-2, a regulator of cholesterol synthesis via HMG-CoA (16). Conversely, hypercholesterolemia has lipotoxic effects on the pituitary-thyroid axis, evidenced in mice by thyroid follicular cell damage, elevated serum/pituitary cholesterol and TSH, and altered pituitary cytoarchitecture after high-cholesterol feeding (17, 18). Despite these insights, the relationship between abnormal lipid metabolism and thyroid function in elderly MDD patients is still unclear.

This study aimed to address the following: (1) the prevalence of thyroid dysfunction in elderly, first-episode, drug-naïve MDD patients with comorbid dyslipidemia; (2) its association with clinical features; and (3) the independent associated factors for thyroid dysfunction in this population.

## 2 Materials and methods

### 2.1 Subjects

This cross-sectional investigation, conducted from 2015 to 2017, enrolled 1,029 participants. The inclusion criteria comprised: (1) Han Chinese ethnicity aged 50–65 years; (2) MDD diagnosis confirmed through the Structured Clinical Interview for DSM-IV (SCID) administered by two qualified psychiatrists; (3)no history of psychotropic medication exposure; and (4) initial depressive episode with HAMD-17 scores ≥24. Exclusion criteria encompassed: (1) comorbid Axis I disorders; (2) significant medical conditions (e.g., active infections, organ failure, malignancies, or traumatic injuries); (3) All participants were assessed using the manic/hypomanic episode module of the SCID to rule out bipolar disorder. Special attention was paid to screening for late-onset bipolar II disorder, ensuring no history of manic or hypomanic episodes. (4)substance use disorders excluding nicotine dependence; and (5) pregnancy or lactation(included as a standard ethical criterion, though highly unlikely in this age group).

The Institutional Review Board of the First Hospital of Shanxi Medical University (2016-Y27) approved this study, which adhered to Declaration of Helsinki principles. All participants provided written informed consent.

### 2.2 Clinical measures

A self-developed questionnaire was administered to collect sociodemographic and clinical data, including age, age at onset, sex, marital status, educational attainment, illness duration (in months), and body mass index (BMI). BMI was calculated using the standard formula: weight in kilograms divided by the square of height in meters (kg/m²).

We employed the 17-item Hamilton Rating Scale for Depression (HAMD) (19) for symptom assessment. The scale incorporates eight five-point (0-4) and nine three-point (0-2) items, with all items graded from none (0) to severe (maximum score per item)).

Anxiety symptoms were measured using the 14-item Hamilton Anxiety Rating Scale (HAMA), with each item rated on a 5-point scale from 0 (none) to 4 (severe) (20). Participants obtaining a total score ≥29 were designated as having severe anxiety symptoms (21).

The Positive and Negative Syndrome Scale (PANSS) positive subscale was employed to evaluate psychotic symptoms in participants (19). This instrument utilizes a 7-point Likert scale where each item is rated from 1 (absent) to 7 (extremely severe). A cutoff score of 15 or higher on this subscale was established as the threshold for determining the presence of psychotic symptoms (22, 23).

Prior to study initiation, two evaluators underwent concurrent training in the administration of all aforementioned assessment instruments to ensure methodological consistency and reliability. Both assessors remained blinded to patients’ clinical status throughout the evaluation process.

### 2.3 Biochemical measurements

Fasting venous blood samples were collected from all participants during the 7:00-9:00 AM period and promptly delivered to the hospital’s clinical laboratory. Both thyroid function and lipid profiles were analyzed the same morning. The thyroid panel comprised thyroid-stimulating hormone (TSH), free triiodothyronine (FT3), free thyroxine (FT4), anti-thyroglobulin antibody (TG-Ab), and anti-thyroid peroxidase antibody (TPO-Ab). The lipid profile included triglycerides (TG), total cholesterol (TC), high-density lipoprotein cholesterol (HDL-C), and low-density lipoprotein cholesterol (LDL-C).

Participants meeting any of the following diagnostic thresholds were classified as having abnormal lipid metabolism: TC ≥5.2 mmol/L, TG ≥1.7 mmol/L, LDL-C ≥3.4 mmol/L, or HDL-C <1.0 mmol/L (24, 25). Abnormal thyroid function (ATF) was defined as TSH >4.2 mIU/L.

### 2.4 Statistical analysis

The distribution of continuous variables was examined using the Kolmogorov-Smirnov one-sample test. The independent samples t-test was applied to continuous data with a normal distribution. For abnormally distributed continuous data, the Mann-Whitney U test was utilized. The Chi-square test was employed to compare categorical data between the two groups. The Bonferroni correction was applied to adjust for multiple testing. To investigate associated factors for abnormal thyroid function in FEDN MDD patients with comorbid ALM, ANOVA was conducted on those with and without abnormal thyroid function. Factors that were significantly different were then included in logistic regression. The area under the receiver operating characteristic curve (AUC-ROC) was used to assess the discriminatory ability of significant parameters in distinguishing patients with abnormal thyroid function from those without.

All data were analyzed using Social Sciences for Windows (SPSS) (version 25.0, developed by IBM in Chicago, IL, USA). Graphs were subsequently plotted using GraphPad Prism version 6.0. Coefficient values, odds ratios (OR), and 95% confidence intervals (CI) were utilized to quantify the strength of association. Two-tailed P values less than 0.05 were considered statistically significant.

## 3 Results

### 3.1 Prevalence and clinical characteristics of abnormal lipid metabolism in elderly first-episode, drug-naïve major depressive disorder patients with and without abnormal lipid metabolism

This study enrolled a total of 1,029 participants. The prevalence of abnormal lipid metabolism among elderly MDD patients was 81.83% (842/1,029).

As indicated in **Table 1**, elderly patients with Major Depressive Disorder (MDD) and lipid metabolism abnormalities exhibited higher Body Mass Index (BMI), Hamilton Depression Rating Scale (HAMD) scores, Hamilton Anxiety Rating Scale (HAMA) scores, Positive and Negative Syndrome Scale (PANSS) positive subscale scores, Thyroid Stimulating Hormone (TSH) levels, Thyroglobulin Antibody (TG-Ab) levels, and fasting blood glucose levels compared to patients without lipid metabolism abnormalities. Binary logistic regression analysis revealed that TSH levels (odds ratio = 1.244, 95% CI = 1.137–1.362, Wald = 6.450, P = 0.000) and HAMD scores (odds ratio = 1.131, 95% CI = 1.028–1.243, Wald= 22.423, P = 0.011) were associated factors for abnormal lipid metabolism.

**Table 1:** Comparison of demographic and clinical characteristics between elderly adult FEDN MDD patients with and without abnormal lipid metabolism.

Variable	MDD with abnormal lipid metabolism	MDD without abnormal lipid metabolism	χ^2^ or F	p
Sample size	842 (81.8%)	187 (18.2%)		
Age (years)	58.67 ± 3.29	56.94 ± 5.38	6.628	0.01
Sex (female, %)	583(69.2%)	128(68.5%)	0.045	0.832
Education (%)			1034.188	0.000
Primary school	313(37.2%)	63(33.7%)		
High school	351(41.7%)	74(40.0%)		
University	136(16.2%)	42(22.5%)		
Master’s	42(4.9%)	8(3.8%)		
Marital status (married, %)	819(97.3%)	175(93.6%)	1041.363	0.000
Suicide attempts, n (%)	196(23.3%)	27(14.4%)	7.043	0.008
Severe anxiety, n (%)	115(13.7%)	15(8.0%)	4.404	0.036
Exhibiting psychotic symptoms, n (%)	99(11.8%)	9(4.8%)	7.856	0.005
Duration of disease, month	7.59 ± 5.33	6.23 ± 4.91	10.376	0.001
BMI (kg/m^2^)	24.51 ± 1.86	24.09 ± 1.53	8.452	0.004
HAMA	21.16 ± 3.53	19.88 ± 3.26	20.44	0.000
HAMD	30.80± 2.84	28.58± 2.67	96.25	0.000
PANSS(P sub-scale)	9.19 ± 4.72	7.75 ± 3.18	15.734	0.000
Fasting blood glucose (mmol/L)	5.46 ± 0.64	5.19 ± 0.64	26.487	0.000
TSH	5.51 ± 2.56	3.63 ± 2.13	87.204	0.000
FT3	4.88 ± 0.72	4.79 ± 0.69	2.346	0.126
FT4	16.63± 3.01	16.53± 3.36	0.176	0.675
A-TG (IU/ml)	103.61 ± 9.49	56.58 ± 7.49	5.287	0.022
A-TPO (IU/ml)	80.34 ± 6.08	56.09 ± 11.01	3.038	0.082

Data are presented as mean ± standard deviation or frequency (percentage); P-values are adjusted using the Bonferroni correction

BMI, body mass index; HAMD, Hamilton Rating Scale for Depression; HAMA, Hamilton Anxiety Rating Scale; PANSS (P sub-scale), positive sub-scale of the Positive and Negative Syndrome Scale; TSH, thyroid stimulating hormone; A-TG, anti-thyroglobulin antibody; A-TPO, anti-thyroid peroxidase antibody; FT3, free triiodothyronine; FT4, free tetraiodothyronine.

### 3.2 Clinical characteristics and biochemical parameters of major depressive disorder with comorbid ALM, with and without abnormal thyroid function

As indicated in **Table 2**, analysis of variance (ANOVA) disclosed significant disparities in demographic and clinical attributes between patients with and without thyroid dysfunction. These differences encompassed the duration of illness, instances of suicide attempts, severe anxiety, psychotic symptoms, Hamilton Depression Rating Scale (HAMD) scores, Hamilton Anxiety Rating Scale (HAMA) scores, Positive and Negative Syndrome Scale (PANSS) positive subscale scores, body mass index (BMI), and fasting blood glucose levels (all at P < 0.05). Conversely, no significant variations were observed regarding age, gender, educational attainment, and marital status (P > 0.05).

**Table 2:** Socio-demographics and clinical characteristics between MDD comorbid ALM with and without abnormal thyroid function.

Variable	MDD with abnormal lipid metabolism (n = 842)			
	NTF(n =261)	ATF (n = 581)	χ^2^ or F	p
Age (years)	57.27 ± 4.32	58.85 ± 4.28	0.871	0.351
Sex (female, %)	179(68.6%)	404(69.5)	0.77	0.782
Education (%)			1.315	0.726
Primary school	98(37.5%)	215(37.0%)		
High school	108(41.4%)	243(41.8%)		
University	45(17.2%)	91(15.7%)		
Master’s	10(3.8%)	32(5.5%)		
married, (%)	248(95.0%)	571(98.3%)	7.202	0.007
Suicide, n (%)	39(14.9%)	157(27.0%)	14.715	0.000
Severe anxiety, n (%)	20(7.7%)	95(16.4%)	11.528	0.001
Exhibiting psychotic symptoms, n (%)	18(6.9%)	81(13.9%)	8.615	0.003
Duration of disease, month	5.97 ± 5.07	8.32 ± 5.28	36.609	0.000
BMI (kg/m^2^)	24.08 ± 1.72	24.70 ± 1.89	20.742	0.000
HAMA	20.59 ± 3.15	21.41 ± 3.66	9.941	0.002
HAMD	29.36± 2.27	31.45± 2.67	110.661	0.000
PANSS(P sub-scale)	8.16 ± 3.58	9.65 ± 5.09	18.187	0.000
Fasting blood glucose (mmol/L)	5.12 ± 0.56	5.62 ± 0.62	124.085	0.000
TC	4.95 ± 0.95	5.80 ± 0.99	135.736	0.000
HDL-C	1.31 ± 0.25	1.14 ± 0.30	60.860	0.000
TG	2.40 ± 0.95	2.37 ± 0.98	0.254	0.615
LDL-C	2.76 ± 0.77	3.33 ± 0.84	85.831	0.000
TSH	2.57 ± 0.99	6.84 ± 1.86	1217.806	0.000
FT3	4.85 ± 0.66	4.90 ± 0.74	0.625	0.429
FT4	16.75 ± 3.06	16.58 ± 2.98	0.602	0.438

Data are presented as mean ± standard deviation or frequency (percentage); P-values are adjusted using the Bonferroni correction

### 3.3 Associated factors for abnormal thyroid function in ALM in patients with MDD

**Figure 1:**
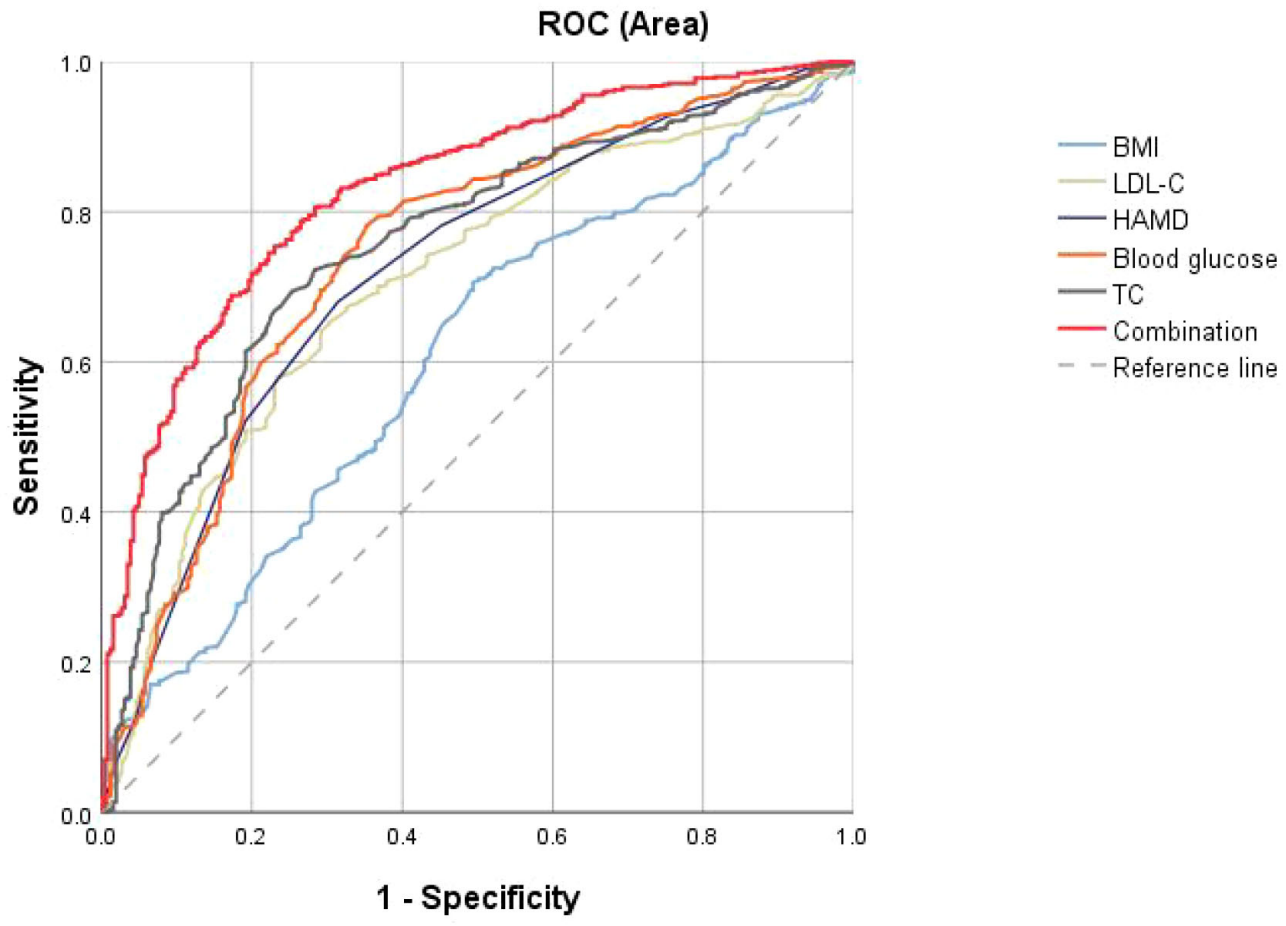
The discriminatory capacity of related factors for distinguishing between patients with and without abnormal thyroid function in MDD comorbid with ALM. The discriminatory capacity of these factors, as measured by AUC-ROC, was 0.755 for TC, 0.743 for blood glucose, 0.721 for HAMD, 0.707 for LDL-C, and 0.605 for BMI. 0.831 for the combination. ROC: receiver operating characteristic. HAMD: Hamilton Rating Scale for Depression.

We subsequently focused on identifying risk factors for abnormal thyroid function in MDD patients with comorbid ALM. Variables that demonstrated significant differences in univariate analysis were subsequently advanced to multivariate logistic regression. As summarized in **Table 3**, the final model identified five independent associated factors: elevated BMI (OR = 1.186, 95% CI = 1.080-1.302), higher HAMD scores (OR = 1.201, 95% CI = 1.116-1.291), increased blood glucose (OR = 4.03, 95% CI = 2.906-5.59), elevated TC (OR = 1.683, 95% CI = 1.340-2.113), and raised LDL-C (OR = 1.299, 95% CI = 1.011-1.679) (all P < 0.05). Conversely, age remained non-significant (OR = 1.00, 95% CI = 0.98-1.02, P = 0.738). The discriminatory capacity of these factors, as measured by AUC-ROC, was 0.755 for TC, 0.743 for blood glucose, 0.721 for HAMD, 0.605 for BMI, and 0.707 for LDL-C. Then we combined parameters with an AUC≥ 0.7, an AUC of 0.831 was observed to differentiate between patients with abnormal and normal thyroid function in MDD patients with comorbid ALM (P < 0.001, 95% CI = 0.767–0.837) (**Figure 1**).

**Table 3:** The associated factors of abnormal thyroid function in MDD patients with ALM.

Variable	B	Wald statistics	df	Sig.	OR	95%CI lower	95%CI upper
Age	0.004	0.112	1	0.738	1	0.98	1.03
HAMD	0.183	24.271	1	0	1.20	1.12	1.29
BMI	0.170	12.807	1	0	1.19	1.08	1.30
LDL-C	0.262	4.170	1	0.04	1.30	1.01	1.67
TC	0.520	20.056	1	0	1.69	1.34	2.11
blood glucose	1.394	69.771	1	0	4.03	2.91	5.59

## 4 Discussion

### 4.1 A summary of the key study findings

To our knowledge, this pioneering study provides the first comprehensive investigation of the relationship between abnormal lipid metabolism and thyroid dysfunction in elderly Chinese patients with first-episode, drug-naïve (FEDN) major depressive disorder (MDD). Our key findings demonstrate that: (1) the prevalence of abnormal thyroid function reaches 69.0% in this specific patient population with comorbid abnormal lipid metabolism (ALM); and (2) multiple metabolic and clinical indicators—including elevated BMI, higher HAMD scores, increased blood glucose, elevated total cholesterol (TC), and raised LDL-C constitute independent associated factors for thyroid dysfunction. These findings collectively indicate that specific clinical features and metabolic abnormalities may contribute to the concurrent development of thyroid dysfunction in MDD patients with ALM.

### 4.2 Comparisons with existing literature

Our investigation revealed a 69.0% prevalence of comorbid abnormal thyroid function in FEDN MDD patients with ALM—significantly higher than in those without ALM. While previous studies have documented complex relationships between thyroid function and both ALM and mood disorders, none have specifically examined thyroid function in FEDN MDD patients with ALM. Contextualizing our findings, Cui et al. reported a 52.2% rate of thyroid dysfunction in general MDD patients (26), while our previous work found substantially different rates between bipolar disorder subgroups: 27% in first-episode mania/hypomania patients versus 60% in those with initial depressive episodes (27). These discrepancies may be attributed to several factors: disease severity and comorbid conditions in MDD patients—particularly concurrent lipid metabolic abnormalities—likely influence thyroid homeostasis, while the metabolic effects of TSH may vary depending on the specific signaling pathways involved.

### 4.3 Possible mechanisms/hypotheses underlying the findings

We demonstrated that MDD patients with ALM and comorbid abnormal thyroid function exhibited higher rates of suicide attempts, anxiety, and psychiatric symptoms, as well as elevated HAMA, HAMD, and PANSS scores. The relationship between thyroid autoimmunity and depression has been documented in previous literature, though with divergent results. While Siegmann et al. (28) projected that over 20% of patients with autoimmune hypothyroidism would develop depression annually suggesting a substantial association. Bode et al. (29) conversely found no statistically significant link between thyroid autoimmunity (primarily based on TPO antibody status) and depression. However, there were no significant differences in social demographic characteristics, such as gender, age, age of onset, level of formal education, and marital status, between the normal and abnormal thyroid function groups among FEDN patients with comorbid MDD and ALM.

Our findings demonstrate that elderly MDD patients with lipid metabolism abnormalities exhibit significantly elevated BMI, TSH, and fasting glucose levels compared to those without such metabolic disturbances. Serum thyroid hormone levels are intricately linked to lipid metabolism, insulin metabolism, and inflammatory factors (30). Patients with autoimmune thyroid disease are more likely to experience abnormal lipid metabolism (30–33). This clinical observation aligns with emerging evidence suggesting crosstalk between adipocyte/hepatocyte-derived metabolic regulators and thyroid function (34). The pathophysiological landscape appears to involve low-grade inflammation as a key mediator - a well-established trigger for depressive episodes in obese individuals (35–37). This inflammatory state operates through dual pathways: it not only induces neuroinflammatory processes that lead to structural and functional neuronal alterations underlying depression (36), but also interacts with HPA axis dysregulation. The consequent glucocorticoid receptor activation promotes consumption of palatable, energy-dense foods as a compensatory mechanism for stress-induced negative affect (38), creating a self-perpetuating cycle that exacerbates weight gain and metabolic dysfunction. Moreover, the association of co-morbidity abnormal thyroid function (ATF) with abnormal glucose in never-treated MDD patients may be attributed to the altered HPT axis. HPT axis triggers autonomic dysfunction. Regardless of changes in thyroid hormone concentrations, fluctuations in blood glucose have a direct effect on TSH secretion (39). But this is only our speculation and needs further verification.

### 4.4 Strengths and weaknesses

This study possesses several notable strengths. First, the large sample size (n = 1,029) of carefully characterized older patients with first-episode, drug-naïve MDD is a key asset. This design effectively minimizes the confounding effects of illness chronicity and psychotropic medications (particularly antidepressants and mood stabilizers) on both metabolic parameters and thyroid function, providing a clearer picture of the intrinsic relationships at the early stage of illness.

Second, our assessment was comprehensive and multidimensional. We concurrently evaluated clinical psychopathology (using standardized rating scales for depression, anxiety, and psychotic symptoms), thyroid function (including hormones and autoantibodies), and a detailed lipid profile. This integrated approach allows for a more holistic understanding of the metabolic-endocrine-psychiatric interface in this population.

Third, methodological rigor was maintained throughout. Diagnoses were confirmed using the Structured Clinical Interview for DSM-IV (SCID) by trained psychiatrists, and all biochemical assays were performed in a standardized manner. Our statistical approach was appropriate for the research questions.

Finally, the primary contribution of this work is its specific focus. To our knowledge, it extends existing knowledge by systematically investigating the prevalence and correlates of thyroid dysfunction within the clinically relevant subgroup of elderly, first-episode, medication-free MDD patients who already have comorbid dyslipidemia. By providing detailed data on this specific phenotypic clustering, our study offers an incremental but important addition to the literature, helping to characterize a common and clinically significant comorbidity profile in treatment-naïve patients.

This study has several methodological limitations that warrant consideration. First, the cross-sectional design precludes causal inference regarding the relationship between abnormal lipid metabolism and thyroid function in elderly FEDN MDD patients; prospective cohort studies are needed to establish temporal sequence. Second, the exclusive recruitment of outpatient populations limits the generalizability of our findings to other clinical settings. Third, we were unable to fully account for several potential confounders, including lifestyle factors (e.g., dietary patterns, physical activity levels) and medication effects that may influence lipid metabolism. Future investigations should address these methodological constraints through longitudinal designs and more comprehensive covariate assessment. The selection of the age range 50–65 years may limit the direct comparability of our findings with studies using the more conventional criteria of ≥60 or ≥65 years. The failure to systematically assess cognitive function represents a limitation of this study. Future research should incorporate tools such as the MMSE or RBANS. The PANSS was used to systematically assess potential comorbid psychotic symptoms, though its applicability in depressive patients is limited. Future studies are advised to employ more generalizable instruments such as the BPRS. Furthermore, studies have demonstrated that the levels of very low-density lipoprotein cholesterol (VLDL-C) are altered in patients with depression, and this biomarker will be incorporated into subsequent research (40). The definition of thyroid dysfunction relied on biochemical thresholds without incorporating clinical symptoms or ultrasonography, which might underestimate the impact of subclinical thyroid disease. Finally, the absence of a healthy control group in this study indicates that the findings must be considered preliminary in nature, and definitive conclusions cannot be drawn.

## 5 Conclusion

In summary, our study revealed a high prevalence of abnormal thyroid function among elderly FEDN patients with MDD and comorbid ALM, indicating that thyroid dysfunction is quite common during the early acute phase of Chinese MDD cases. Moreover, our research demonstrated that the prevalence of suicide attempts, severe anxiety, and psychotic symptoms was significantly higher in elderly MDD patients with ALM and abnormal thyroid function compared to those without such thyroid dysfunction. Additionally, certain clinical indicators, including blood glucose and lipid levels, may contribute to the co-occurrence of abnormal thyroid function in MDD patients with comorbid ALM. Furthermore, the primary findings of this study have significant clinical implications. Firstly, in the prevention of abnormal thyroid function, MDD patients with comorbid ALM could be regarded as a potential subtype of MDD. Specific treatment strategies should be tailored for this potential subtype. Consequently, in clinical practice, it is recommended that the assessment of thyroid function in elderly MDD patients encompass regularly monitor parameters such as blood glucose and lipid levels to prevent inappropriate treatment decisions.

## Data Availability

The original contributions presented in the study are included in the article/supplementary material. Further inquiries can be directed to the corresponding author.
